# Tumor lysis syndrome in premature infant prompting early resection of a large sacrococcygeal teratoma: a case report

**DOI:** 10.1186/s12887-023-04193-w

**Published:** 2023-09-02

**Authors:** Aditi Dey, Rita Wyrebek, Loraine Torres, Danilo Escoto, Fauzia Shakeel, Jennifer Mayer

**Affiliations:** 1 Maternal Fetal Neonatal Institute, Johns Hopkins All Children’s Hospital, Florida, USA; 2 Department of Obstetrics and Gynecology, Bayfront Medical Center, Orlando Health, Florida, USA; 3 Cancer and Blood Disorders Institute, Johns Hopkins All Children’s Hospital, Florida, USA

**Keywords:** Glucocorticoids, Premature infant, Premature neonates, Rasburicase, Hyperkalemia, Tumor lysis syndrome, Sacrococcygeal teratoma, Neonate

## Abstract

**Background:**

Sacrococcygeal teratomas (SCTs) are the most common congenital neoplasm and often require resection soon after birth. There are rare reports of cardiac arrest during surgery due to manipulation of the tumor triggering secondary necrosis and hyperkalemia.

**Case presentation:**

This case describes a very preterm infant with a SCT who develops spontaneous preoperative tumor lysis syndrome (TLS). The medical team utilized rasburicase and the patient underwent total gross resection at 40 h of life.

**Conclusions:**

We emphasize the importance of the early recognition and management of tumor lysis syndrome in SCT with rasburicase, aggressive management of hyperkalemia and consideration of early resection of SCTs even in the case of a very premature infant.

## Background

Of congenital neoplasms, sacrococcygeal teratomas (SCT) are the most common occurring in 1 out of 40,000 infants with a large female predominance [1]. These lesions typically require surgical resection soon after birth [2]. Survival rates range from 77 to 94% [3]. Poor prognostic factors include prematurity, fetal hydrops, high output cardiac failure, fetal anemia, and rupture of SCT [3].

Tumor lysis syndrome (TLS) is infrequent in patients with solid tumors [4]. There are only 6 case reports published in peer-review journals in the English language upon query of the PubMed Database for SCTs and TLS between the years 1980 and 2022. Our report uniquely highlights the critical timing of resection en bloc of an SCT as well as the safety and efficacy of rasburicase in a premature infant with SCT associated TLS.

### Case presentation

**Fig. 1:**
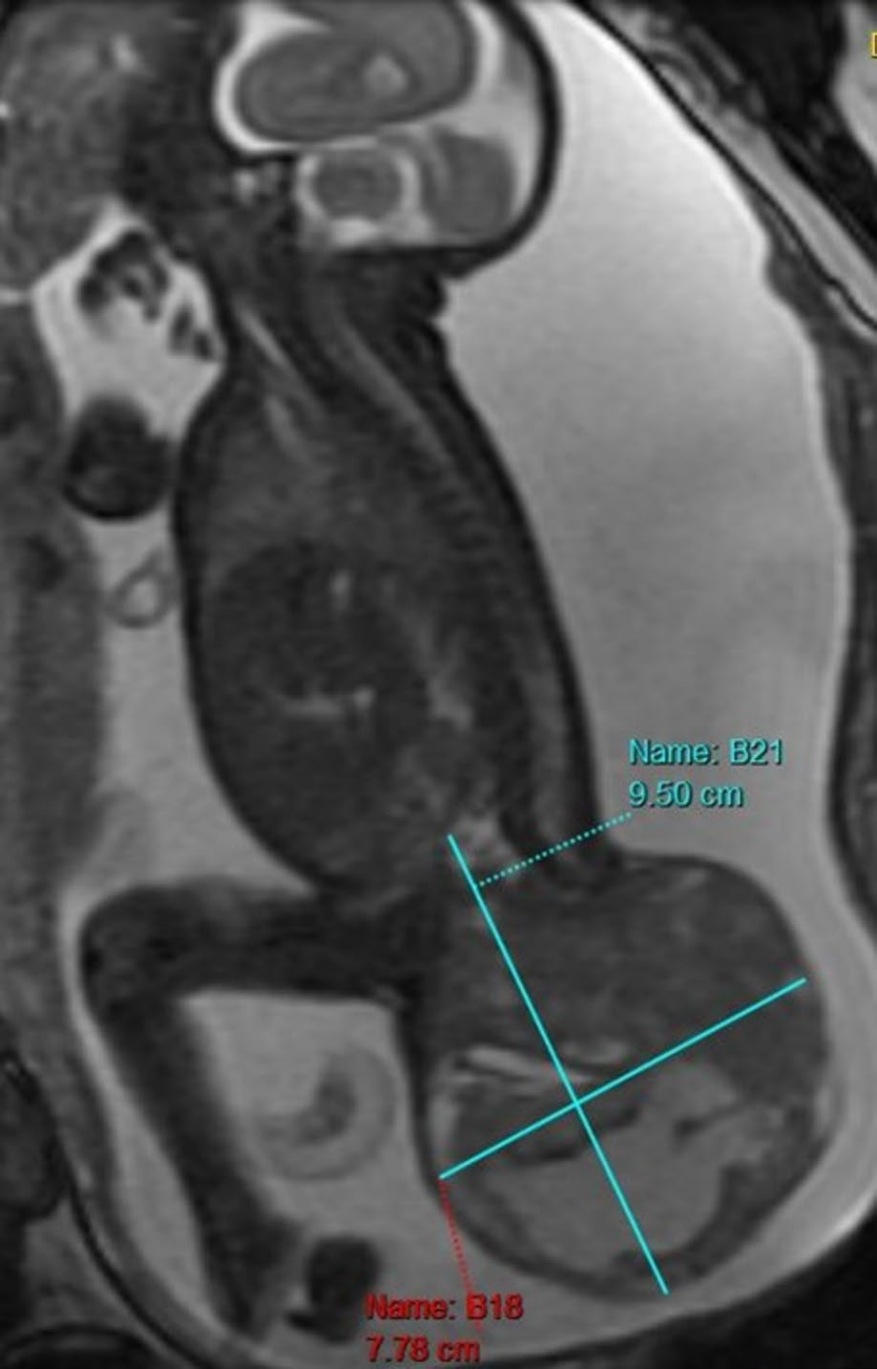
Fetal magnetic resonance image demonstrating a complex sacrococcygeal structure suspicious for a sacrococcygeal teratoma measuring 9.5 cm × 7.78 cm

A previously healthy 34-year-old gravida 5 para 2 female was followed by maternal fetal medicine due to prenatal ultrasound findings concerning for a fetal SCT and placenta previa and accreta. A karyotype was performed during amniocentesis and revealed a 46, XX female. Fetal magnetic resonance imaging (MRI) (Fig. 1) demonstrated a complex mixed structure extending inferiorly from the sacral spine consistent with SCT.

**Fig. 2:**
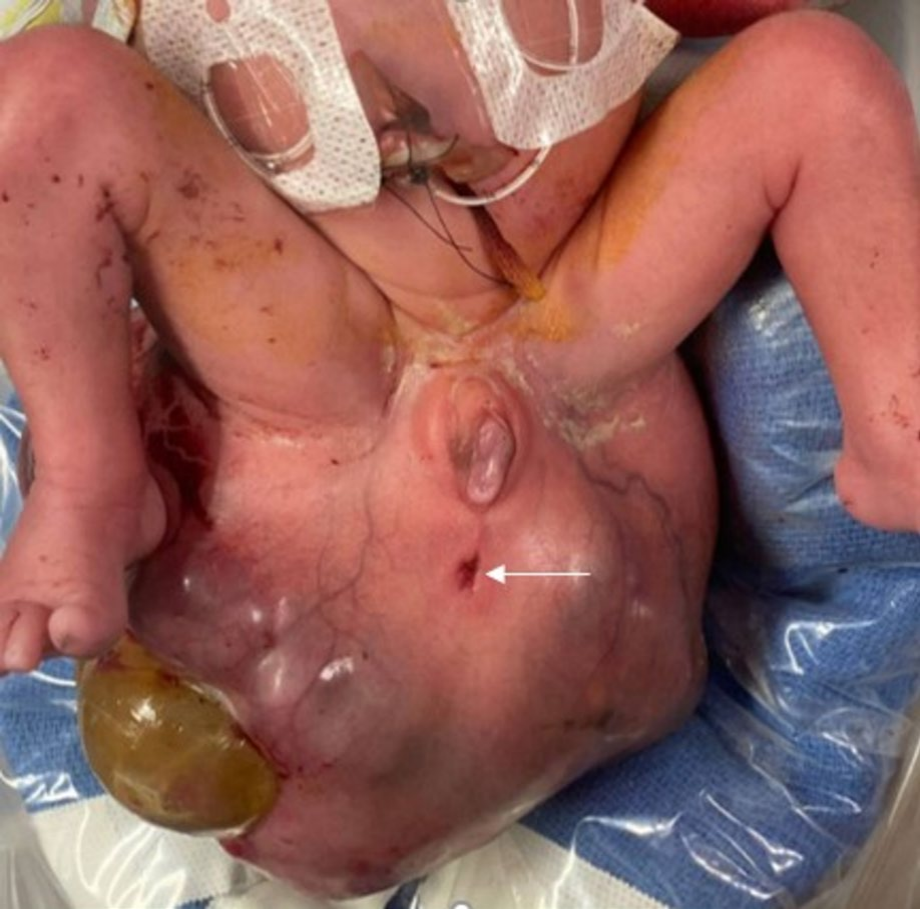
Appearance of the infant soon after delivery with SCT in anterior view with anterior displacement of rectum (white arrow)

The mother presented to the hospital with vaginal bleeding at 26 weeks’ gestation and received prenatal vitamins, betamethasone, and magnesium sulfate. At 29 weeks and 3 days, with worsening contractions and vaginal bleeding, a classical cesarean section and hysterectomy under general anesthesia were performed. A female infant was born weighing 2.4 kg (> 99^th^ percentile on the Fenton Growth Curve) with a massive SCT shown in Fig. 2.

**Fig. 3:**
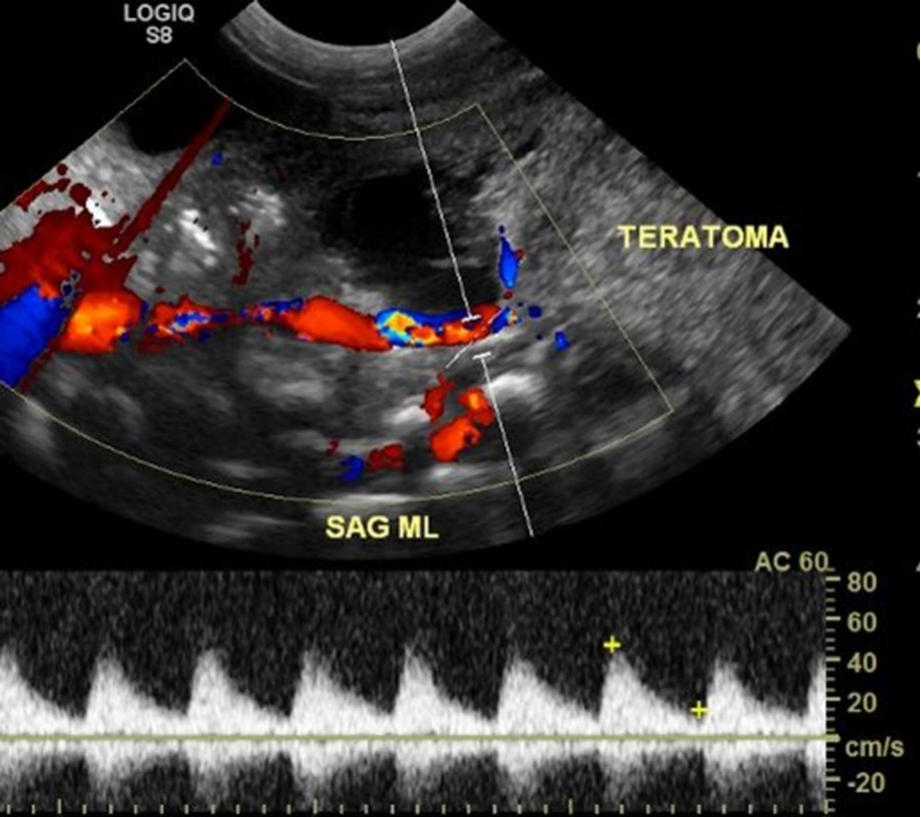
A pre-operative pelvic ultrasound was performed revealing a tiny distal vessel arising from the aorta appearing to be going in the direction of a solid and cystic lesion anterior to the sacrum, thought to be a teratoma

Neonatal resuscitation comprised endotracheal intubation and surfactant administration due to poor respiratory effort and 100% oxygen requirement. Apgar scores were 1, 3 and 6 at 1, 5 and 10 min, respectively. The patient was transferred to the neonatal intensive care unit (NICU) where a pelvic ultrasound was done confirming prenatal findings. The SCT extended to L3/L4 with blood supply from a vessel arising from the aortic bifurcation visualized on pre-operative pelvic ultrasound (Fig. 3).

**Fig. 4:**
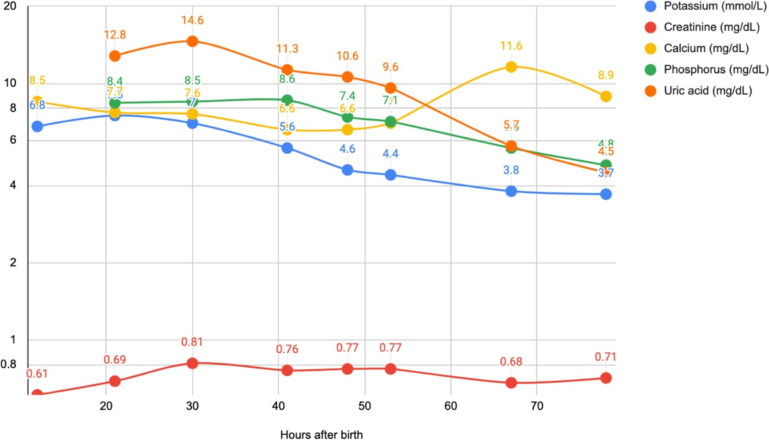
Peri-operative serum laboratory measurements of serum potassium, calcium, uric acid, phosphorus, and creatinine from the time of birth

Though the initial plan was to delay surgery until neonatal transition was completed, the patient developed hyperkalemia within 12 h of life with a serum potassium of 6.8 mmol/L, without any extraneous potassium being administered in the intravenous (IV) fluids. At 24 h of age, the hyperkalemia worsened to 7.5 mmol/L with a stable serum creatinine of 50 µmol/L and urine output. The presence of TLS was confirmed with hyperuricemia of 862 µmol/L (normal 170–770 µmol/L), hypocalcemia, hyperkalemia and worsening renal injury, as demonstrated in Fig. 4. Hyperkalemia was treated with albuterol, furosemide, insulin and dextrose infusion, and calcium gluconate for cardiac stability. One dose of rasburicase (0.1 mg/kg/dose) was administered IV. Clinical examination of the mass did not reveal any gross areas of hemorrhage or necrosis.

**Fig. 5:**
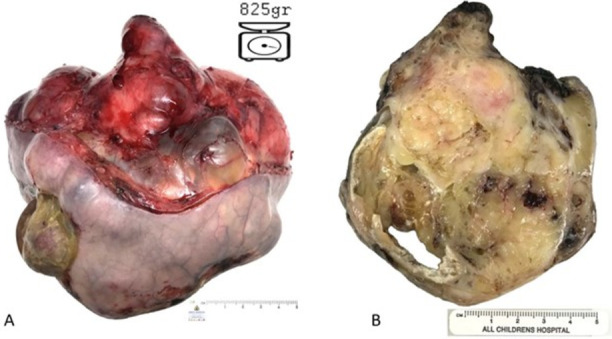
**A** External view of SCT weighing 825 g. **B** Cross section of SCT. On pathology review, the mass was a 13 cm x 12 cm x 10 cm sacrococcygeal teratoma – Immature Grade 3 with microscopic foci of yolk sac (primitive endodermal/endodermal sinus) tumor as a focus of malignancy. Immunohistochemistry demonstrated glial and endodermal elements. Hemosiderin staining on microscopic analysis revealed a tiny remote hemorrhage. No malignant cells were identified

**Fig. 6:**
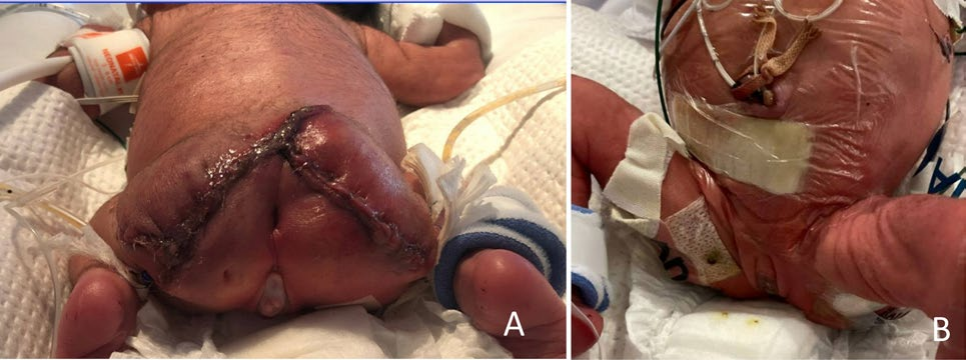
**A** Postoperative Day #2. View of posterior surgical wound. **B** Postoperative Day #2. View of anterior approach. Images demonstrate preservation of anus and external genital structures with reapproximation of musculature and skin

**Fig. 7:**
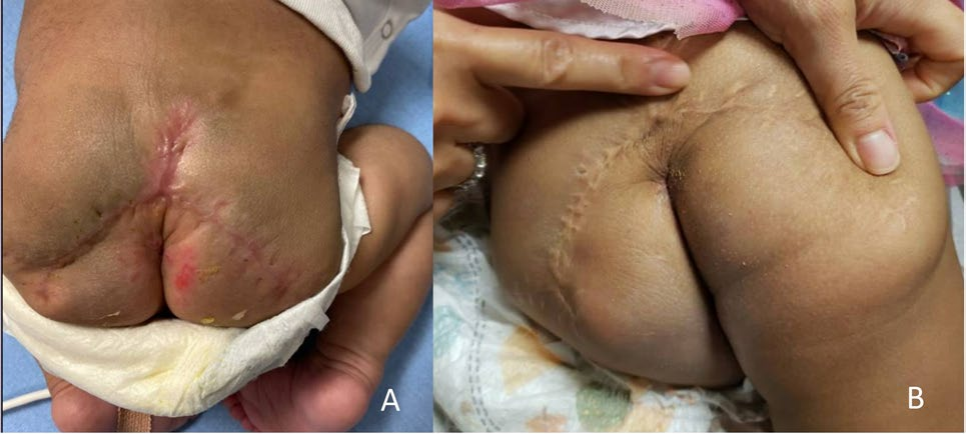
**A** Postoperative Day #60 during outpatient surgical follow up. Mild erythema on buttocks was secondary to diaper dermatitis and **B** 1.5 years post-resection with excellent surgical healing and rectum in normal position

The risks and benefits of surgery and anesthesia were weighed in the setting of a very preterm infant. With diagnosed TLS, the risk of worsening tumor necrosis, and relative control of electrolytes with aggressive medical management, the decision was made to undergo surgical total gross resection of the tumor at about 40 h of life. Anesthesia included fentanyl and rocuronium. Insulin and glucose were continued throughout the procedure for the management of hyperkalemia with close intra-operative potassium monitoring. The middle sacral artery, the vascular source for the teratoma, was ligated. The tumor was resected (shown in Fig. 5) and pelvic floor musculature reconstructed, following which both urethra and rectum were patent (Fig. 6). The uterus was retracted and preserved. A Hegar dilator was inserted into the anus to allow for visualization of the rectal wall. The rectal wall and musculature were spared. The coccyx was divided in order to remove the teratoma noted to have presacral extension. The pelvic floor musculature, subcutaneous tissues, and skin were reapproximated. Intra-abdominal structures were without injury. The serum potassium immediately after resection was 5.6 mmol/L and remained within normal limits thereafter. The patient was extubated on post-operative day 4 with enteral feeds started shortly thereafter. There were multiple genetic studies performed, including the Invitae Pediatric Solid Tumor Panel (negative for 54 genes for variants associated with solid tumors), Fulgent Custom NGS Panel of *NSD1, SENP1, and SETBP1* (negative for significant sequence of copy-number variants) and a karyotype and chromosomal microarray of the sacrococcygeal teratoma (no clinically significant imbalance or loss of heterozygosity observed). A routine head ultrasound was without intraventricular hemorrhage performed in the context of prematurity, extensive resuscitation and surgical needs. Serum alpha-fetoprotein decreased from 124,461.5 ng/mL on the first day of life to 3 ng/mL on follow up at 1.5 years of age. The patient was discharged around 60 days postnatal age with excellent wound healing (Fig. 7).

## Discussions and conclusions

Sacrococcygeal teratomas (SCTs) are the most common congenital neoplasm and often require resection soon after birth. Although the postnatal survival of SCT is high, prenatal mortality ranges between 25–37% [5]. Prematurity and low Apgar scores are poor prognostic factors [2, 5, 6], and upwards of 50% of fetuses with sacrococcygeal teratomas deliver prematurely [7]. Surgical resection remains the mainstay of therapy, though fetal surgery, endoscopic laser ablation, and radiofrequency ablation have been used as treatment [8].

There are only a few case reports detailing hyperkalemia as a complication of SCT with variable survival, especially in the setting of prematurity, and none describing the use of rasburicase. Despite aggressive medical management, hyperkalemia led to the demise of several extremely premature neonates even though surgical resection was attempted, emphasizing the need for early recognition of neonates at risk of TLS and frequent perioperative electrolyte monitoring [4, 6]. In neonates closer to term, early surgery was believed to be therapeutic for worsening or refractory hyperkalemia that was otherwise being medically managed [2] stressing the importance of careful handling of the tumor to avoid necrosis [9].

TLS is a life-threatening constellation of metabolic derangements characterized by hyperuricemia, hyperkalemia, hyperphosphatemia, hypocalcemia leading to acute renal failure. It is common in hematologic malignancies and following the initiation of chemotherapy. Traditionally, most solid tumors are considered low-risk for the development of TLS, however, bulky solid tumors (among those, germ cell tumors) may be considered at intermediate risk [10]. While the typical management of tumor lysis syndrome includes aggressive intravenous hydration in order to preclude renal injury [11], this must be carefully balanced in the very preterm infant who is at particularly high risk of complications of fluid overload including severe chronic lung disease and increased mortality [12]. Rasburicase is a recombinant urate-oxidase enzyme, converting uric acid into an inactive and soluble metabolite, allantoin. There have been reports of the safe use of rasburicase in neonates in the setting of tumor lysis syndrome [13, 14], though there is a risk of death with underlying glucose-6-phosphate-dehydrogenase (G6PD) deficiency [15]. Notably, G6PD testing was not performed prior to the initiation of rasburicase in our patient owing to the long turnaround time of this laboratory test and time-sensitive nature of the treatment at a critical time point to preserve renal function. There was no family history suggestive of G6PD deficiency, the patient was a female, and there were no obvious signs of anemia or hemolysis on peripheral blood smear.

In the pediatric population, TLS has also been described in neuroblastomas, lymphomas and can occur spontaneously or with corticosteroid administration [16]. Steroids are often used as components of chemotherapy due to their lympholytic effect and act by inducing cellular growth arrest and apoptosis [17]. TLS has been reported in patients receiving corticosteroids exclusively, especially in hematologic malignancies [17]. Dexamethasone and betamethasone, both strongly recommended antenatally for fetal lung maturation and improved neonatal outcomes, have been previously associated with TLS. There is one reported case of a term neonate delivered with a neck teratoma and early development of TLS thought to be attributed to antenatal corticosteroid administration [16]. It remains unclear what role antenatal corticosteroid administration played in the development of TLS and further carefully designed studies are needed to establish any relationships, should they exist.

While there is no definitive relationship between tumor size and suspected necrosis, it is conceivable that the larger the tumor grows, it can no longer support angiogenesis centrally as it grows externally, leading to central necrosis and cell death. Therefore, it is possible that the sheer bulk of this child’s SCT was the causative factor for the development of TLS. Additionally, while there is mention of a tiny, remote hemorrhage on microscopic analysis of the tumor, it certainly remains unclear what degree of hemorrhage and resulting tissue anoxia would be capable of activating the cascade of cell death resulting in tumor lysis syndrome.

The timing of surgical intervention warrants careful discussion especially in the very premature infant owing to the risks of surgery and anesthesia. In addition to risks inherent to operating on a fragile, immature neonate with unique thermoregulatory, respiratory and hemodynamic physiology, infants with sacrococcygeal teratomas pose the possibility of intraoperative severe hemorrhage, coagulopathy, and electrolyte disturbances owing to tumor manipulation [2]. Our case demonstrates that in concordance with previous literature, delaying surgical intervention portends the risk of worsening tumor necrosis and resulting hyperkalemia, thus early resection may in fact be life-saving at the onset of tumor lysis syndrome. We emphasize the importance of the early recognition and careful management of SCT-associated tumor lysis syndrome with rasburicase and aggressive management of hyperkalemia as a key to our patient’s survival. Clinicians should maintain a high index of suspicion of tumor lysis syndrome with serial laboratory assessments and multidisciplinary consideration of early resection of SCTs even in the case of a very premature infant.

## Data Availability

Data sharing is not applicable to this article as no datasets were generated or analyzed during the current study.

## Abbreviations

SCT: Sacrococcygeal teratoma
TLS: Tumor lysis syndrome
MRI: Magnetic resonance imaging
NICU: Neonatal intensive care unit
IV: Intravenous

## References

[1] Lahdes-Vasama TT, Korhonen PH, Seppänen JM, Tammela OK, Iber T (2011). Preoperative embolization of giant sacrococcygeal teratoma in a premature newborn. J Pediatr Surg.

[2] Abraham E, Parray T, Ghafoor A (2010). Complications with massive sacrococcygeal tumor resection on a premature neonate. J Anesth.

[3] Tran KM, Flake AW, Kalawadia NV, Maxwell LG, Rehman MA (2008). Emergent excision of a prenatally diagnosed sacrococcygeal teratoma. Paediatr Anaesth.

[4] Jona JZ (1999). Progressive tumor necrosis and lethal hyperkalemia in a neonate with sacrococcygeal teratoma (SCT). J Perinatol.

[5] Makin EC, Hyett J, Ade-Ajayi N, Patel S, Nicolaides K, Davenport M (2006). Outcome of antenatally diagnosed sacrococcygeal teratomas: single-center experience (1993–2004). J Pediatr Surg.

[6] Kim JW, Gwak M, Park JY, Kim HJ, Lee YM (2012). Cardiac arrest during excision of a huge sacrococcygeal teratoma - A report of two cases -. Korean J Anesthesiol.

[7] Holterman AX, Filiatrault D, Lallier M, Youssef S (1998). The natural history of sacrococcygeal teratomas diagnosed through routine obstetric sonogram: a single institution experience. J Pediatr Surg.

[8] Abraham E, Parray T, Ghafoor A (2010). Complications with massive sacrococcygeal tumor resection on a premature neonate. J Anesth.

[9] Reinoso-Barbero F, Sepulveda I, Pérez-Ferrer A, De Andres A (2009). Cardiac arrest secondary to hyperkalemia during surgery for a neonatal giant sacrococcygeal teratoma. Paediatr Anaesth.

[10] Cairo MS, Coiffier B, Reiter A, Younes A (2010). TLS Expert Panel. Recommendations for the evaluation of risk and prophylaxis of tumour lysis syndrome (TLS) in adults and children with malignant diseases: an expert TLS panel consensus. Br J Haematol..

[11] Coiffier B, Altman A, Pui C, Younes A, Cairo MS (2008). Guidelines for the management of pediatric and adult tumor lysis syndrome: an evidence-based review. J Clin Oncol.

[12] Matsushita FY, Krebs VLJ, Ferraro AA, de Carvalho WB (2020). Early fluid overload is associated with mortality and prolonged mechanical ventilation in extremely low birth weight infants. Eur J Pediatr.

[13] Wyrebek R, Mohammad A, Iqbal A, Dighe D, Giordano L. Treatment of metabolic abnormalities with rasburicase in a premature neonate. Arch Clin Cases. 2018;5(2):37–41.

[14] McNutt DM, Holdsworth MT, Wong C, Hanrahan JD, Winter SS (2006). Rasburicase for the management of tumor lysis syndrome in neonates. Ann Pharmacother.

[15] Zaramella P, De Salvia A, Zaninotto M, Baraldi M, Capovilla G, De Leo D (2013). Lethal effect of a single dose of rasburicase in a preterm newborn infant. Pediatrics.

[16] Ponmudi N, Beryl S, Santhanam S, Beck M. Tumour lysis in newborn: Spontaneous or secondary to antenatal steroids?. BMJ Case Rep. 2018:bcr–2017. 10.1136/bcr-2017-223107. 10.1136/bcr-2017-223107 PMC5893956 29618468

[17] Kim JO, Jun DW, Tae HJ, Lee KN, Lee HL, Lee OY (2015). Low-dose steroid-induced tumor lysis syndrome in a hepatocellular carcinoma patient. Clin Mol Hepatol.

